# Improved Kalman Filtering Algorithm Based on Levenberg–Marquart Algorithm in Ultra-Wideband Indoor Positioning

**DOI:** 10.3390/s24227213

**Published:** 2024-11-11

**Authors:** Changping Xie, Xinjian Fang, Xu Yang

**Affiliations:** 1 School of Geomatics, Anhui University of Science and Technology, Huainan 232001, China; 2022201727@aust.edu.cn (C.X.); xyang@aust.edu.cn (X.Y.); 2 The Key Laboratory of Universities in Anhui Province for Prevention of Mine Geological Disasters, Huainan 232001, China

**Keywords:** Kalman filter, UWB, least square method, Levenberg–Marquardt algorithm, positioning algorithm

## Abstract

To improve the current indoor positioning algorithms, which have insufficient positioning accuracy, an ultra-wideband (UWB) positioning algorithm based on the Levenberg–Marquardt algorithm with improved Kalman filtering is proposed. An alternative double-sided two-way ranging (ADS-TWR) algorithm is used to obtain the distance from the UWB tag to each base station and calculate the initial position of the tag by the least squares method. The Levenberg–Marquardt algorithm is used to correct the covariance matrix of the Kalman filter, and the improved Kalman filtering algorithm is used to filter the initial position to obtain the final position of the tag. The feasibility and effectiveness of the algorithm are verified by MATLAB simulation. Finally, the UWB positioning system is constructed, and the improved Kalman filter algorithm is experimentally verified in LOS and NLOS environments. The average X-axis and the Y-axis positioning errors in the LOS environment are 6.9 mm and 5.4 mm, respectively, with a root mean square error of 10.8 mm. The average positioning errors for the X-axis and Y-axis in the NLOS environment are 20.8 mm and 18.0 mm, respectively, while the root mean square error is 28.9 mm. The experimental results show that the improved algorithm has high accuracy and good stability. At the same time, it can effectively improve the convergence speed of the Kalman filter.

## 1. Introduction

With the rapid development of technology, the Internet of Things has become a new trend in social development, and high-precision navigation and positioning are its core technologies related to national security and economic development [1]. The Global Navigation Satellite System (GNSS) can achieve high positioning accuracy in flat and open outdoor areas. Nowadays, GNSS systems based on low-Earth-orbit (LEO) satellites are designed to provide indoor positioning services, but the positioning accuracy can be maintained at around 1 m, which does not satisfy the high-accuracy positioning requirements of indoor positioning algorithms. High-precision indoor navigation and positioning systems are in high demand because of the acceleration of urbanization and the growth of interior scenes. Compared with Bluetooth, wireless fidelity (WIFI), radio frequency (RFID), and other indoor positioning technologies, ultra-wide band (UWB) technology has the advantages of high precision, low power consumption, and strong signal penetration, so is suitable for high-precision indoor positioning [2,3]. UWB positioning equipment mainly consists of tags and base stations [4]. Distance information between tags and base stations is calculated by measuring the propagation time of pulse signals between them. By triangulating three or more distance measurements, the specific position of the tag can be determined [5,6]. However, due to design issues in hardware systems and the limitations of practical conditions, errors such as hardware device errors, non-line-of-sight environment errors, and multipath effects occur during the calculation process, leading to decreased positioning accuracy. Therefore, it is necessary to develop a high-precision algorithm to meet the growing demand for indoor positioning.

Scholars have conducted a substantial amount of research in the field of UWB positioning. Che et al. [7] discussed the application of indoor positioning systems (IPSs) using ultra-wideband (UWB) technology in the industrial Internet of Things. The article explains the unique characteristics and challenges of UWB technology and evaluates the advantages and limitations of using machine learning algorithms for UWB positioning. FLORIO et al. [8] proposed an all-digital simultaneous angle-of-arrival (AOA) estimation architecture based on phase interferometry. The AOA algorithm can use fewer base stations to complete localization. Still, the localization accuracy is greatly affected when the tags are far away from the base stations or when the signals are obstructed and not applicable in complex indoor environments. Sang et al. [9] analyzed five algorithms for UWB positioning: trilateration, the least squares method, the first-order Taylor series iteration method, the extended Kalman filter (EKF) algorithm, and the unscented Kalman filter (UKF) algorithm. Addressing the shortcomings of the above algorithms, Yang et al. [10] adopted the time-of- flight (TOF) ranging method and optimized it using a Kalman filter algorithm to improve positioning accuracy. Still, this method has limited application scenarios and cannot achieve high-precision positioning results. Yang et al. [11] used time-of-flight (TOF) ranging to establish positioning equations, proposed the weighted centroid algorithm to calculate the initial position of tags, and introduced residual weighting and Newton iteration to obtain the optimal position of the tags. However, this algorithm may lead to large positioning errors when the tag position is at the boundary of the measurement range. Yang et al. [12] proposed a dynamic path loss parameter measurement method based on the center of mass by measuring the path loss parameter in the smallest neighborhood area where the node to be measured is located to improve the accuracy of the received signal strength indication (RSSI).

Due to the complexity of real environments, the accuracy of UWB positioning algorithms is affected by various environmental factors, among which non-line-of-sight (NLOS) error is the most common. Many scholars have researched reducing the impact of NLOS on positioning accuracy. There are two main approaches. The first one uses high-precision positioning algorithms to reduce positioning errors in the NLOS state. Fang and Yang et al. [13,14] addressed the problem of the standard time deviation in UWB devices due to environmental interference and proposed an improved TOF algorithm. They also proposed an adaptive robust Kalman filter (ARKF) algorithm for indoor scenarios where NLOS interference exists. This algorithm can effectively improve the accuracy and stability of UWB positioning, reduce positioning errors, and achieve good positioning results. De Cock et al. [15] discussed methods to address occlusion issues in UWB indoor positioning systems. They proposed an occlusion mitigation strategy based on the relative direction of the body and tags to the UWB base station, composed of Gaussian mixture models (GMMs), selecting appropriate GMMs based on the estimated relative direction. Position calculation uses inertial measurement units (IMUs) on tags and UWB tracking algorithms. Liu et al. [16] introduced the robust particle filter (RPF) based on robust theory into UWB positioning, which can significantly avoid the positioning errors caused by truncation and NLOS errors. The other identifies the line-of-sight (LOS)\NLOS state through the algorithm and compensates for the data in the NLOS state to improve the positioning accuracy of the algorithm. Li et al. [17] proposed an improved strong tracking Kalman filter (ISTCKF) for application in UWB positioning systems. This algorithm identifies NLOS observations by reconstructing observed values, utilizing statistical properties of observation noise and NLOS errors, and calculating attenuation factors based on the identification results to mitigate major positioning errors and obtain accurate positioning results. Zhang et al. [18] analyzed the factors and characteristics of NLOS formation in indoor environments, established an anchor point LOS/NLOS information map using prior information, and designed a robust adaptive extended Kalman filter algorithm based on the anchor point LOS/NLOS information map, which effectively reduces the impact of NLOS on positioning accuracy. Li et al. [19] determined the data containing NLOS errors by calculating the standard deviation of the squared residual between the predicted and measured values and compensating for these data through compensation filtering. Finally, the Kalman filter (KF) algorithm determines the tag’s position. With the rapid development of machine learning algorithms, more and more scholars are integrating machine learning algorithms into UWB positioning algorithms. Gu et al. [20] proposed combining neural network algorithms with a self-adjusting Kalman filter algorithm, using the positioning results obtained by neural network algorithms as the initial values for the Kalman filter algorithm. This algorithm has the advantages of high accuracy, good real-time performance, and stability. Cui et al. [21] addressed the issue of the low accuracy of UWB positioning by proposing a UWB wireless ranging algorithm that combines wavelet packet decomposition and long short-term memory (LSTM). Tian et al. [22] proposed a KF-LSTM algorithm that combines KF with LSTM neural networks. It first processes UWB data using a KF to reduce noise, then inputs the data into the LSTM network for training, utilizing LSTM’s ability to handle time-series features to obtain more accurate tag positions. Gao et al. [23] integrated two machine learning algorithms, CNN and SVM, with a UWB hybrid positioning algorithm, the Chan–Taylor–CNN–SVM algorithm (C-T-CNN-SVM), achieving high-precision positioning results. It combines the SVM-based signal classification method, CNN-based signal recognition and error elimination method, and Chan–Taylor-based hybrid weighted positioning algorithm to obtain accurate positioning results. Lu et al. [24] proposed an adaptive path recognition method based on artificial neural networks, which can identify NLOS in different environments.

In conclusion, most of the current UWB positioning algorithms use improved algorithms based on the KF. Still, there is a general lack of high positioning accuracy, while the algorithm’s structure is more complex, resulting in the algorithm’s shortcomings in real time. Some algorithms have been combined with deep learning algorithms, which need a large number of training samples to obtain a better model, which leads to the positioning results fluctuating greatly when faced with complex and changeable indoor environments. Based on the above analysis, and considering that the actual environment is prone to change and the iterative nature of the KF observation equation is similar to that of the Gauss–Newton method for finding the function extremum, we chose to improve the KF by using the Levenberg–Marquardt algorithm, which is a nonlinear optimization algorithm with fast convergence, high stability, and strong adaptability. At the same time, the covariance matrix in the KF process includes the state covariance matrix and the measurement noise covariance matrix, representing the estimation uncertainty. In the UWB localization process, a change in the environment has a large impact on the magnitude of the measurement noise, which leads to a decrease in the accuracy of the localization algorithm. Therefore, it was chosen to adopt the Levenberg–Marquardt algorithm. The covariance matrix pair correction reduces the uncertainty of the estimation and makes the estimation closer to the real value.

In summary, this paper proposes a UWB positioning algorithm based on Levenberg–Marquardt’s improved KF. The algorithm uses the least squares method to calculate the position and obtain the tag’s initial coordinates. The Levenberg–Marquardt algorithm is used to improve the KF, and the tag’s initial coordinates are used as the improved KF’s initial value. Finally, the positioning algorithm is verified by simulations and experiments. The remainder of this article is structured as follows: Section 2 introduces the principles related to the algorithm in question. Section 3 explains improving the Kalman filter using the Levenberg–Marquardt method model. Section 4 outlines our simulation experiments and real environment experiments, with a result analysis. Section 5 discusses the results and outlines future outlooks. Section 6 concludes this paper.

## 2. Principles of UWB Positioning with Improved Kalman Filtering

### 2.1. Alternative Double-Sided Two-Way Ranging

**Figure 1:**
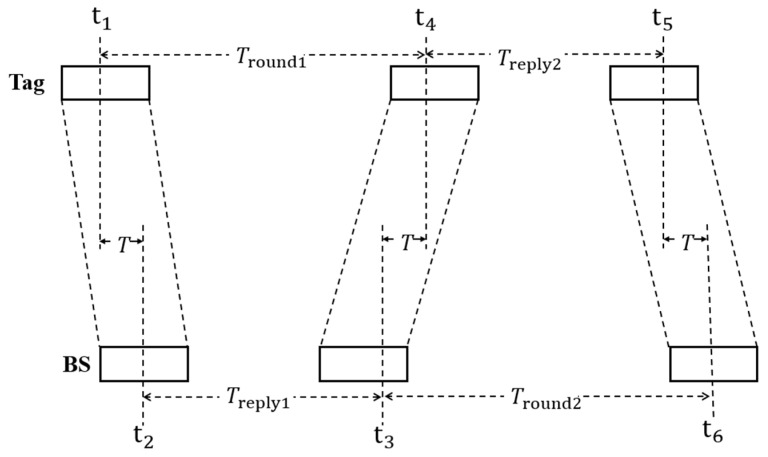
Schematic diagram of the asymmetric bidirectional bilateral ranking algorithm.

The ADS-TWR method [25], which uses polling to determine the distance between three UWB base stations and the tag, is used in this article. Firstly, the tag records the exact instant at which it transmits a ranging request signal to the base stations. When the base stations within its communication range receive the ranging request signal, the moment it is received is recorded. Subsequently, the base stations send a feedback signal to the tag, and the moments when the base stations send the feedback signal and when the tag receives the feedback signal are recorded. Finally, the tag transmits the ranging request signal to the base stations once again. The time stamps of the tag’s second ranging request signal send, and the base stations’ second ranging request signal receive are logged. The ADS-TWR algorithm’s basic idea is depicted in Figure 1.

The time from the tag to the base station (T) can be calculated using Equations (1)–(5):

(1) $$T_{round1}=t_{4}-t_{1}$$

(2) $$T_{round2}=t_{6}-t_{3}$$

(3) $$T_{reply1}=t_{3}-t_{2}$$

(4) $$T_{reply2}=t_{5}-t_{4}$$

(5) $$T=\frac{T_{round1}\times T_{round2}-T_{reply1}\times T_{reply2}}{\left(T_{round1}+T_{round2}+T_{reply1}+T_{reply2}\right)}$$

In the formulas, $t_{1}$ represents the instant at which the tag sends the base station its initial range request signal; $t_{2}$ represents the instant the ranging request signal is received by the base station; $t_{3}$ represents the moment when the base station sends the feedback signal; $t_{4}$ represents the instant the tag receives the feedback signal; $t_{5}$ represents the instant the tag delivers its second ranging request signal; $t_{6}$ represents the moment when the base station receives the ranging request signal for the second time; T indicates the propagation time of the pulse signals between the tags and base stations.

The T signals between the UWB tags and base stations can be used to determine the distance between the tag and the base station using Equation (6):

(6) d=T×c

In the formula, d is the distance between the tag and the base station; c is the speed of light.

### 2.2. The Least Squares Method

The least squares method (LSM) is a mathematical optimization algorithm. It obtains the fitting result of the data by minimizing the square of the errors [26]. The process of solving unknown variables using the LSM is relatively simple, and the obtained results minimize the sum of squares of errors between the actual data and fitting results. The principle of using the LSM to solve UWB positioning coordinates is as follows:

In two-dimensional space, the distance between the base station and the tag can be represented as

(7) $$d_{i}^{2}={\left(x_{i}-x\right)}^{2}+{\left(y_{i}-y\right)}^{2}$$

In the equation, $d_{i}$ represents the distance between the tag and the ith base station, $\left(x_{i},y_{i}\right)$ represents the x axis and the y axis coordinates of the ith base station, and (x,y) represents the x-axis and the y-axis coordinates of the tag.

This paper uses four base stations to calculate the tag position. Thus, Equation (7) can be rewritten as Equation (8):

(8) $$\left\{\begin{array}{l} d_{1}^{2}={\left(x_{1}-x\right)}^{2}+{\left(y_{1}-y\right)}^{2}\\ d_{2}^{2}={\left(x_{2}-x\right)}^{2}+{\left(y_{2}-y\right)}^{2}\\ d_{3}^{2}={\left(x_{3}-x\right)}^{2}+{\left(y_{3}-y\right)}^{2}\\ d_{4}^{2}={\left(x_{4}-x\right)}^{2}+{\left(y_{4}-y\right)}^{2} \end{array}\right.$$

Sequentially subtracting the first equation from the second, third, and fourth equations in Equation (8) and simplifying, the resulting system of equations can be written in matrix form as

(9) AX=b

In the equation,

$$A=\left[\begin{array}{c} x_{2}-x_{1}\;y_{2}-y_{1}\\ x_{3}-x_{1}\;y_{3}-y_{1}\\ x_{4}-x_{1}\;y_{4}-y_{1} \end{array}\right],X={\left[\begin{array}{cc} x & y \end{array}\right]}^{T},\;b=\frac{1}{2}\left[\begin{array}{c} d_{1}^{2}-d_{2}^{2}+(x_{2}^{2}+y_{2}^{2})-(x_{1}^{2}+y_{1}^{2})\\ d_{1}^{2}-d_{3}^{2}+(x_{3}^{2}+y_{3}^{2})-(x_{1}^{2}+y_{1}^{2})\\ d_{1}^{2}-d_{4}^{2}+(x_{4}^{2}+y_{4}^{2})-(x_{1}^{2}+y_{1}^{2}) \end{array}\right]$$

Let the error vector be δ, which can be written as

(10) δ=AX−b

When the error vector δ tends to zero, the X has an optimal solution, denoted as f, as shown in Equation (11):

(11) $$f=\delta^{2}=\delta^{T}\delta ={\left(AX-b\right)}^{T}(AX-b)$$

Taking the derivative of Equation (11) and setting it equal to zero, we obtain

(12) $$\frac{df(X)}{dx}=2A^{T}AX-2A^{T}b=0$$

If $A^{T}A$ is a nonsingular matrix, then AX=b has a unique solution, which can be solved using Equation (13):

(13) $$\;X={\left(A^{T}A\right)}^{-1}A^{T}b$$

The obtained X is the coordinates of the tag.

### 2.3. Improving the Kalman Filtering Algorithm

Like the LSM, the Kalman filtering algorithm (KF) is a linear algorithm. Compared to the LSM, the KF has better denoising effects. The principle of the KF is to minimize the variance of state errors when seeking the optimal solution, thereby obtaining a better estimation of the state [27]. When using the KF to solve the coordinates of UWB tags, a linear Kalman state observation model of discrete time-invariant systems can be introduced, as shown in Equation (14):

(14) $$\left\{\begin{array}{l} x_{k}=Ax_{k-1}+Bu_{k-1}+w_{k-1},w_{k}\sim N(0,Q)\\ z_{k}=Hx_{k}+v_{k},v_{k}\sim N(0,R) \end{array}\right.$$

where $x_{k}$ represents the state variable at time step k, A represents the state transition matrix, $u_{k}$ represents the input variable at time step k, B is the input control matrix, $z_{k}$ is the measurement vector, H is the transition matrix from state to measurement, $w_{k}$ and $v_{k}$, respectively, represent process noise and measurement noise, Q and R, respectively, denote the variances of process noise and measurement noise.

This paper computes the position of the UWB localization tags in a two-dimensional plane, with the observed state variables including the coordinates of the localization tags in the plane, as well as their corresponding velocities and accelerations, thus defining the state variables, as shown in Equation (15):

(15) $$x_{k}={\left(\left[\begin{array}{cccccc} x & y & \dot{x} & \dot{y} & \ddot{x} & \ddot{y} \end{array}\right]\right)}^{T}$$

In this equation, x and y represent the coordinates of the localization tags in the x axis and the y axis in the two-dimensional plane; $\dot{x}$ and $\dot{y}$ represent the velocities of the localization tags in the x axis and the y axis; $\ddot{x}$ and $\ddot{y}$ represent the accelerations of the localization tags on the x axis and the y axis.

Define the state transition matrix A, the state-to-measurement transition matrix H, the variance of process noise Q, and the variance of measurement noise R as Equation (16):

(16) $$A=\left[\begin{array}{cccccc} 1 & 0 & T & 0 & T^{2}/2 & 0\\ 0 & 1 & 0 & T & 0 & T^{2}/2\\ 0 & 0 & 1 & 0 & T & 0\\ 0 & 0 & 0 & 1 & T & 0\\ 0 & 0 & 0 & 0 & 1 & 0\\ 0 & 0 & 0 & 0 & 0 & 1 \end{array}\right],H=\left[\begin{array}{cccccc} 1 & 0 & 0 & 0 & 0 & 0\\ 0 & 1 & 0 & 0 & 0 & 0 \end{array}\right],\;Q=diag\left(\left[T^{4}/4\;\;T^{4}/4\;\;T^{2}/2\;\;T^{2}/2\;\;\;\;1\;\;\;\;1\right]\right),R=diag\left(\left[\begin{array}{cc} \sigma_{\nu x}^{2} & \sigma_{\nu y}^{2} \end{array}\right]\right)$$

In the equation, T represents the system update rate, $\sigma_{vx}^{2}$ and $\sigma_{vy}^{2}$ represent the measurement noise on the x and y axes, respectively, Q and R can be derived based on observations.

The KF follows the steps below:

(17) $${\hat{x}}_{k}^{-}=A{\hat{x}}_{k-1}+Bu_{k-1}$$

(18) $$P_{k}^{-}=AP_{k-1}A^{T}+Q$$

(19) $$K_{k}=\frac{P_{k}^{-}H^{T}}{HP_{k}^{-}H^{T}+R}$$

(20) $${\hat{x}}_{k}={\hat{x}}_{k}^{-}+K_{k}(z_{k}-H{\hat{x}}_{k}^{-})$$

(21) $$P_{k}=(I-K_{k}H)P_{k}^{-}$$

In the equation, $P_{k}^{-}$ and $P_{k}$ are the prior and posterior estimates of the error covariance matrix.

Due to the iterative nature of the observation equations, similar to the Gauss–Newton method of finding the extreme points of a function, the performance is often unstable. When the covariance estimate is too low, the state observation does not guarantee a reduction in the estimation error [28]. Therefore, this paper uses the Levenberg–Marquardt algorithm [29] to improve the KF. The Levenberg–Marquardt algorithm is one of the most widely used nonlinear optimization algorithms. It combines the advantages of the Gauss–Newton method and the gradient descent method, and it has global and local fast convergence.

The basic theory of the Levenberg–Marquardt algorithm is a first-order Taylor expansion of a nonlinear function:

(22) f(x+Δx)≈f(x)+J(x)Δx

In this equation, J(x) is the derivative of f(x) with respect to x. The Levenberg–Marquardt algorithm finds a vector, such that $∥f(x+\Delta x){∥}^{2}$ is minimized, which can be obtained by substituting into Equation (22):

(23) $$\underset{\Delta x}{\min}\frac{1}{2}||f(x)+J(x)\Delta x{\left|\right|}^{2}$$

This is obtained by deriving Equation (23) for Δx and adding the damping coefficient μ:

(24) $$\Delta x=-{\left[J{\left(x\right)}^{T}J(x)+\mu I\right]}^{-1}J{\left(x\right)}^{T}f(x)$$

where μ is generally assigned empirically and adjusted for practical effects.

To overcome the issue where the observation update of the state cannot guarantee a consistent reduction in the estimation error due to the iterative nature of the Kalman filter observation equations and where the estimate of the covariance array is lower than the true value, the Levenberg–Marquardt algorithm is used to correct $P_{k}^{-}$ during each iteration, as shown in Equation (25). Then, an iterative observation update is carried out with the corrected $P_{k}^{-}$.

(25) $$P_{k}^{-}=\left[I-P_{k}^{-}{\left(P_{k}^{-}+\frac{1}{\mu }I\right)}^{-1}\right]P_{k}^{-}$$

### 2.4. Contrast Experiments

Among the existing UWB positioning algorithms, the trilateration algorithm (TA) [30], LSM, KF, and UKF have better positioning results. To verify the feasibility of the proposed localization algorithm, the KF algorithm using the Levenberg–Marquardt (LMKF) method proposed in this paper is compared with the TA, LSM, KF, and UKF. The X-axis error, the Y-axis error, the average positioning error (AVE), and the RMSE of the different algorithms’ positioning accuracies were compared through simulation experiments [31], and the AVR and RMSE were calculated as follows:

(26) $$AVE=\frac{1}{n}\sum_{i=1}^{n}\left(\left|x_{i}-x\right|\right)$$

(27) $$RMSE=\sqrt{\frac{1}{n}\sum_{i=1}^{n}\left[{\left(x-x_{i}\right)}^{2}+{\left(y-y_{i}\right)}^{2}\right]}$$

In this equation, $\left(x_{i},y_{i}\right)$ represents the calculated coordinates of the tag for the ith iteration.

#### 2.4.1. Trilateration Algorithm

The trilateration algorithm is the most direct and fundamental of the UWB positioning algorithms; it is a traditional method based on geometric techniques. In a two-dimensional plane, the tag’s position can be obtained by calculating the intersection points of three circles with known radii. Suppose the positions of the three UWB base stations are A1(0,0), $A2(x_{2},0)$, and $A3(x_{3},y_{3})$, the position of the tag can be calculated using Pythagorean theorem:

(28) $$d_{1}^{2}=x^{2}+y^{2}$$

(29) $$d_{2}^{2}={\left(x_{2}-x\right)}^{2}+y^{2}$$

(30) $$d_{3}^{2}={\left(x_{3}-x\right)}^{2}+{\left(y_{3}-y\right)}^{2}$$

In this equation, $d_{i}$ represents the distance between the ith base station and the tag. Subtracting Equation (28) from Equation (29) yields

(31) $$x=\frac{d_{1}^{2}-d_{2}^{2}+x_{2}^{2}}{2\cdot x_{2}}$$

Subtracting Equation (28) from Equation (30) yields

(32) $$y=\frac{d_{1}^{2}-d_{3}^{2}+x_{3}^{2}+y_{3}^{2}-2xx_{3}}{2y_{3}}$$

Substituting Equation (31) into Equation (32) yields

(33) $$y=\frac{(d_{1}^{2}-d_{3}^{2}+x_{3}^{2}+y_{3}^{2})x_{2}-(d_{1}^{2}-d_{2}^{2}+x_{2}^{2})x_{3}}{2y_{3}x_{2}}$$

Equations (31) and (33) can be used to obtain the tag’s coordinates in the two-dimensional plane.

#### 2.4.2. Unscented Kalman Filter Algorithm

Since the KF algorithm only applies to linear systems, the estimation effect of nonlinear systems is very poor. It cannot be used in practical engineering applications, so the UKF algorithm for nonlinear systems is derived. The UKF essentially approximates the Gaussian filtering of nonlinear system functions and estimates the mean and covariance of the state of the nonlinear system by using a deterministic sample sampling method based on the unscented transform (UT). The mean and covariance of the state of the system are estimated to obtain the posterior distribution of the state. The UKF process is as follows:

First, define the nonlinear system at moment k as

(34) $$\left\{\begin{array}{l} x_{k}=f(x_{k-1})+\omega_{k}\\ z_{k}=h(x_{k})+v_{k} \end{array}\right.$$

In this equation, f is a nonlinear equation of state; h is the nonlinear observation equation.

The UT process is

(35) $$\left\{\begin{array}{l} X^{\left(0\right)}={\bar{X}}_{n}t=0\\ X^{\left(0\right)}=\bar{X}+(\sqrt{(n+\lambda )P}),i=1\;\sim \;n\\ X^{\left(0\right)}=\bar{X}-(\sqrt{(n+\lambda )P}),i=n+1\;\sim \;2n \end{array}\right.$$

(36) $$\left\{\begin{array}{l} \omega_{m}^{\left(0\right)}=\frac{\lambda }{n+\lambda }\\ \omega_{c}^{\left(0\right)}=\frac{\lambda }{n+\lambda }+(1-\alpha^{2}+\beta )\\ \omega_{m}^{\left(i\right)}=\omega_{c}^{\left(i\right)}=\frac{\lambda }{2(n+\lambda )},i=1\;\sim \;2n \end{array}\right.$$

In this equation, n is the dimension of the state vector, $\bar{X}$ is the mean value of the state vector, P is the variance, ${\left(\sqrt{P}\right)}_{i}$ denotes the ith column of the root of the matrix variance, m is the mean, c is the covariance, the superscript in the formula denotes the ith sampling point, $\lambda =a^{2}(n+k)-n$ is a scalable scale parameter, which can reduce the prediction error, α controls the distribution state of the sampling point, and k denotes a parameter to be selected, which is chosen to ensure that (n+λ)P is a semipositive definite matrix. β is generally non-negative and adjusts the accuracy of the covariance. The parameters can be selected according to experience.

Using the estimates of the state quantities and variances under the current calendar element, a time update is performed to estimate the state quantities and variances for the next calendar element:

(37) $$x_{k-1}^{i}=\left[{\hat{x}}_{k-1}{\hat{x}}_{k-1}+\sqrt{(n+\lambda )P_{k-1}}{\hat{x}}_{k-1}-\sqrt{(n+\lambda )P_{k-1}}\right]$$

(38) $$x_{k/k-1}^{i}=f(x_{k-1}^{i})$$

(39) $${\hat{x}}_{k/k-1}=\sum_{i=0}^{2n}\omega_{m}^{i}x_{k/k-1}^{i}$$

(40) $$P_{kk-1}=\sum_{i=0}^{2n}\omega_{m}^{i}(x_{k/k-1}^{i}-{\hat{x}}_{k/k-1}){\left(x_{k/k-1}^{i}-{\hat{x}}_{k/k-1}\right)}^{T}+Q$$

(41) $$x_{klk-1}^{i}=\left[{\hat{x}}_{klk-1}{\hat{x}}_{klk-1}+\sqrt{(n+\lambda )P_{klk-1}}{\hat{x}}_{klk-1}-\sqrt{(n+\lambda )P_{klk-1}}\right]$$

Perform measurement updates:

(42) $$z_{k/k-1}^{i}=h(x_{k/k-1}^{i})i=0,1,\cdots ,2n$$

(43) $${\hat{z}}_{kk-1}=\sum_{i=0}^{2n}\omega_{m}^{i}z_{k/k-1}^{i}$$

(44) $$P_{zz}=\sum_{i=0}^{2n}\omega_{m}^{i}(z_{k/k-1}^{i}-{\hat{z}}_{k/k-1}){\left(z_{k/k-1}^{i}-{\hat{z}}_{k/k-1}\right)}^{T}+R$$

(45) $$P_{xz}=\sum_{i=0}^{2n}\omega_{m}^{i}(x_{k/k-1}^{i}-{\hat{z}}_{k/k-1}){\left(x_{k/k-1}^{i}-{\hat{z}}_{k/k-1}\right)}^{T}$$

Calculate the Kalman gain and update the system state and covariance:

(46) $$K_{k}=P_{xz}P_{zz}^{-1}$$

(47) $${\hat{x}}_{k}={\hat{x}}_{k/k-1}+K_{k}(z_{k}-{\hat{z}}_{k/k-1})$$

(48) $$P_{k}=P_{k/k-1}-K_{k}P_{zz}K_{k}^{T}$$

## 3. Improving the Kalman Filter Using the Levenberg–Marquardt Method for the UWB Localization Algorithm Model

**Figure 2:**
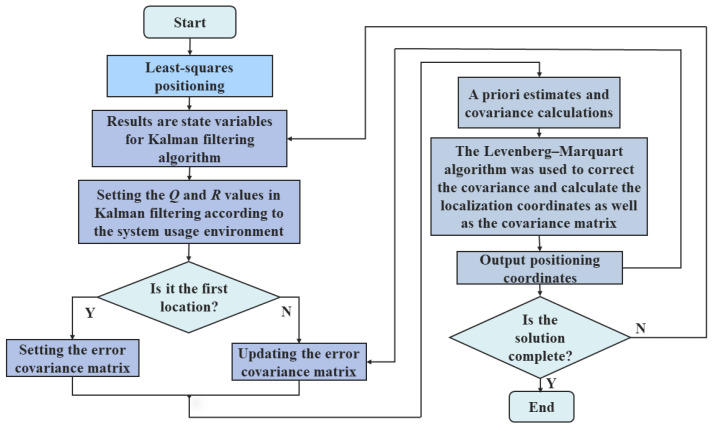
Flowchart of the model.

The LSM convergence speed is faster, and the KF algorithm has a better denoising effect, which can obtain more accurate localization results; the Levenberg–Marquardt algorithm can correct the covariance matrix of KF to improve the positioning accuracy further. Based on the above analysis, this paper takes the localization result obtained by the LSM as the initial position of the label, improves the KF using the Levenberg–Marquardt algorithm, and applies the LMKF to the initial position of the label, which can obtain a localization result with high accuracy. The flowchart of the algorithm is shown in Figure 2.

Firstly, the ADS–TWR algorithm is used to obtain the distance between the UWB base station and the tag; LSM is used to calculate the initial position of the tag; and its position coordinates are used as the state variables for LMKF filtering. The system noise covariance and observation noise covariance are set according to the environmental factors. Then, the a priori estimate and covariance matrix are calculated. At the same time, the Levenberg–Marquardt algorithm is used to correct the covariance, and the damping coefficient μ is introduced to improve the convergence and stability of the algorithm so that the estimate is closer to the actual value. Subsequently, the state and covariance are updated, while the stage’s positioning coordinates are output to complete the data calculation.

## 4. Experimental Analysis

### 4.1. Simulation Experiment and Analysis

This study conducted simulations in the MATLAB environment, assuming that the tag coordinates remained unchanged. A four-anchor, one-tag positioning system was selected, with anchor coordinates of A(0,0), B(100,0), C(100,100), and D(0,100); the true coordinates of the tag were (50,50). The sampling time t was 10 s, and the sampling interval was 0.1. The tag received and recorded signals from each anchor, and the channel noise followed $N(0,\delta^{2})$.

#### 4.1.1. Performance Analysis of Different Localization Algorithms

**Figure 3:**
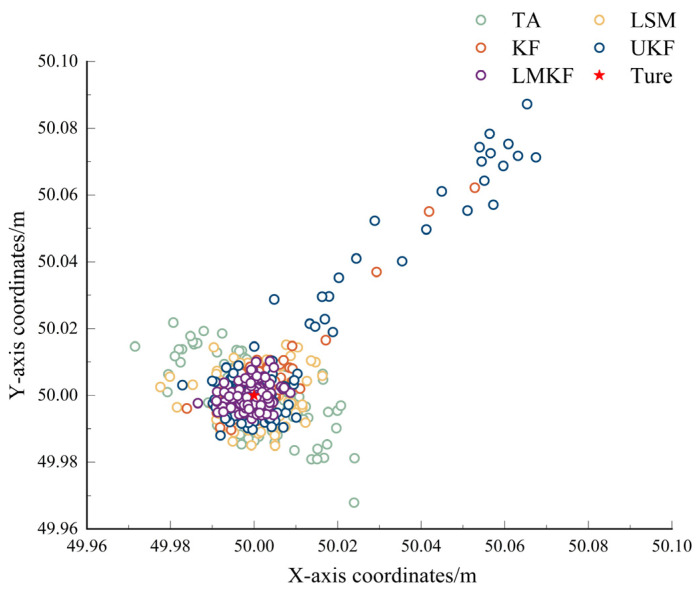
Simulated positioning results of different algorithms.

**Figure 4:**
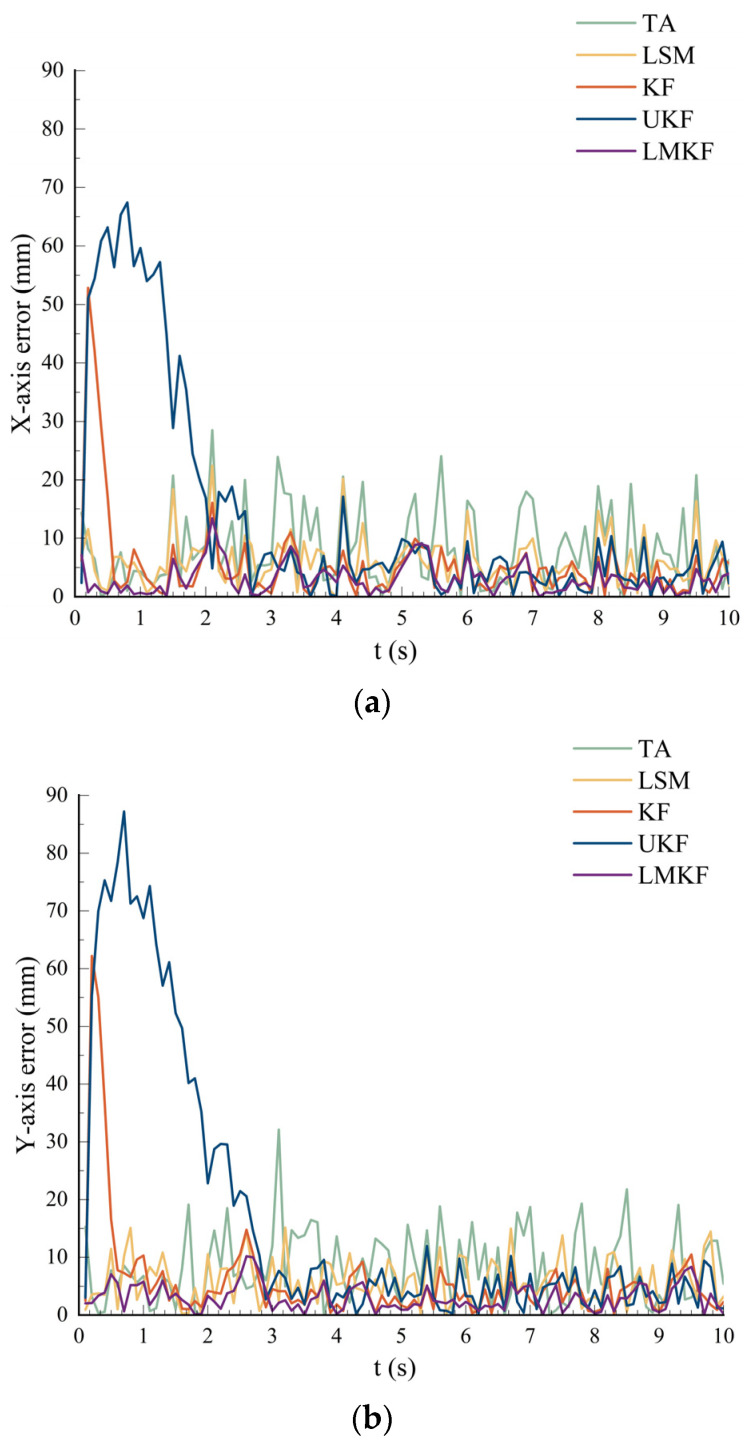
(**a**) Comparison of the X-axis error; (**b**) comparison of the Y-axis error.

To compare the positioning accuracy of the five localization algorithms, the real coordinates of the tag were set to (50,50), and the channel noise was set to 0.01. The simulated distance information was used for calculation with the TA, LSM, KF, UKF, and LMKF algorithms, and the positioning results and errors are shown in Figure 3 and Figure 4.

Figure 3 and Figure 4 demonstrate that while all five positioning algorithms were capable of producing positioning results that were quite accurate in static environments, the LMKF algorithm proposed in this paper had significantly fewer positioning errors than TA and LSM. The LMKF, KF, and UKF had similar accuracies in the later stages of the experiment. The LMKF algorithm effectively compensated for the slow convergence speed of the KF and UKF in the initial stage. Table 1 displays several algorithms’ AVE and RMSE.

As shown in Table 1, under the LOS environment with a noise of 0.01, the LMKF could control the AVE in the X-axis and the Y-axis directions within about 3 mm. Compared with the TA, LSM, KF, and UKF, the AVE in the X-axis direction was reduced by 62.90%, 49.50%, 42.60%, and 76.89%, respectively, while that in the Y-axis direction was reduced by 63.68%, 49.66%, 47.96%, and 81.70%, respectively; RMSE was reduced by 62.90%, 46.86%, 60.44%, and 84.90%. The experimental results showed that the LMKF has high accuracy and stability.

#### 4.1.2. Performance Analysis of Localization Algorithms Under Different Noise Conditions

**Figure 5:**
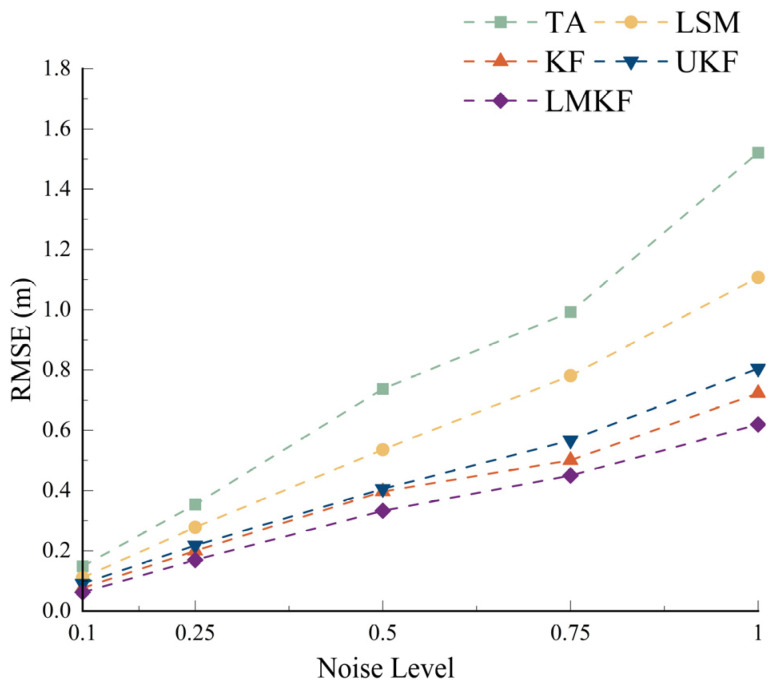
Location performance of the five algorithms under different noise levels.

To compare the positioning performance of the different algorithms under different noise conditions, the true coordinates of the tag were set to (50,50), and the channel noise levels were set to 0.1, 0.5, 0.75, and 1. The simulated distance information was used for the calculation with the TA, LSM, KF, UKF, and LMKF algorithms. The positioning accuracies of the different algorithms are shown in Figure 5.

Figure 5 shows that the localization error of the LMKF rises with the increase in system noise. Still, the localization errors under different noise levels are smaller than those of the TA, LSM, KF, and UKF. Meanwhile, the experimental results showed that the LMKF is insensitive to noise. Compared with the TA, LSM, and UKF, the RMSE was reduced by 54.71%, 42.43%, 10.16%, and 20.69%, respectively, when the noise variance was 0.75. When the noise variance was 1, the RMSE is reduced by 59.29%, 44.07%, 14.34%, and 23.09%, respectively. The LMKF performed well in high-noise environments so can be applied in severe noise environments.

### 4.2. Experimental Data and Analysis

#### 4.2.1. Hardware

**Figure 6:**
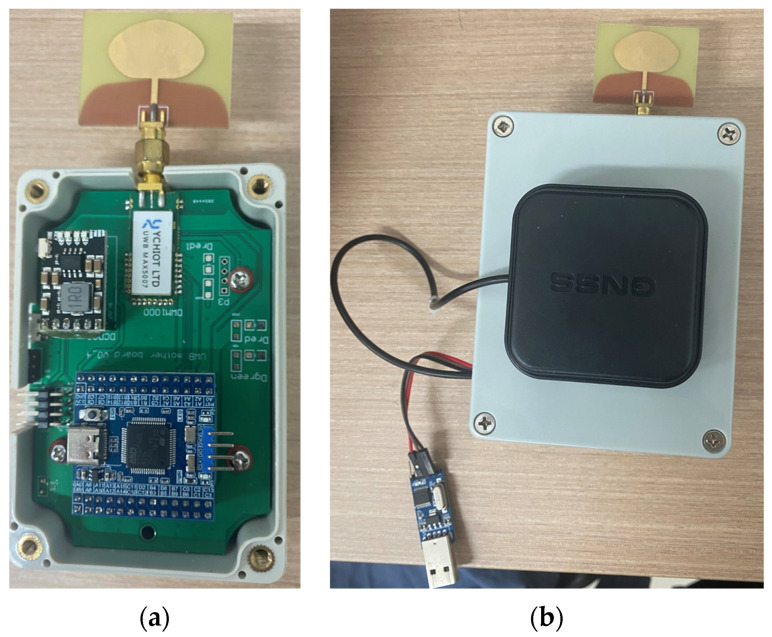
(**a**) UWB positioning base station; (**b**) UWB positioning tag.

This paper adopted the Haida Xingyu H1-U1-type UWB networking positioning system produced by Hi-Target from China; the equipment is shown in Figure 6, and the specific parameters are shown in Table 2. The device had a built-in Advantech’s MAX5007 UWB positioning module, which was based on decaWave’s DWT000 chip design of an ultra-wideband transmitter module, integrating an antenna, RF circuits, power management, and clock circuits. It could be used in the TWR or TDOA positioning system, with a coverage radius of 1000 m, a ranging error of less than 10 cm, and a positioning error of less than 15 cm. The device could obtain information about the distance from the tag to the base station and use it as initial information for the subsequent positioning process.

#### 4.2.2. Performance Analysis of Localization Algorithm in LOS Environment

**Figure 7:**
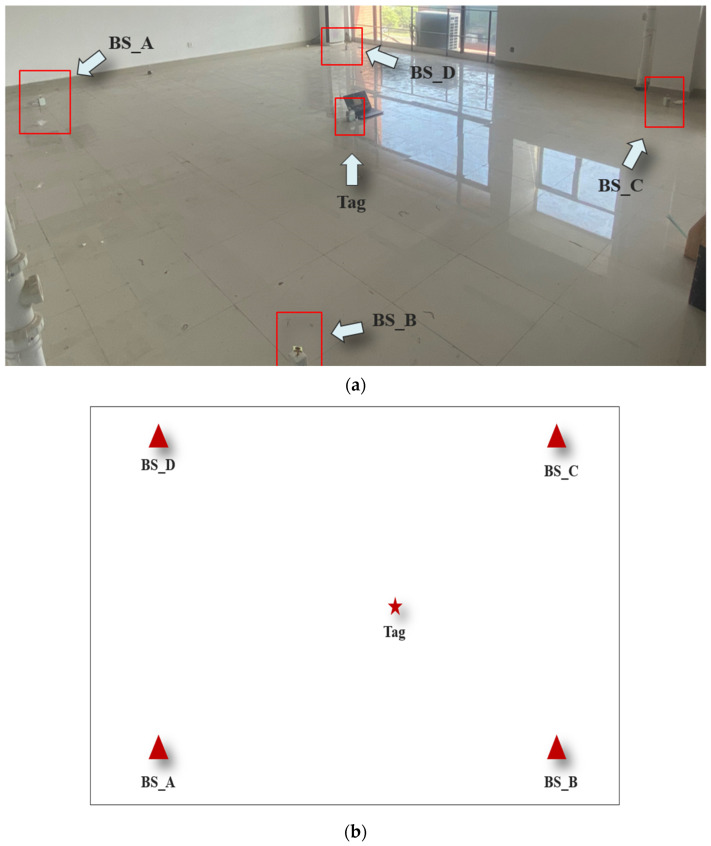
(**a**) Experimental setup diagram; (**b**) UWB base station and tag distribution.

**Figure 8:**
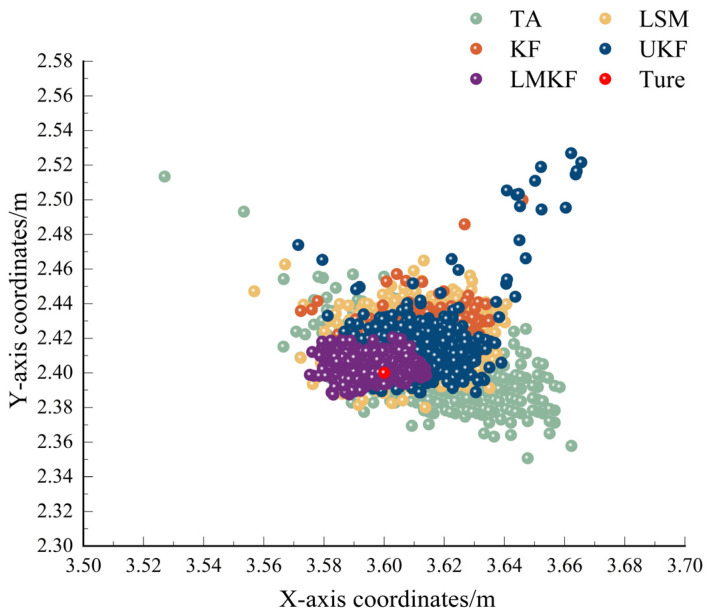
Positioning scatterplot in LOS environment.

**Figure 9:**
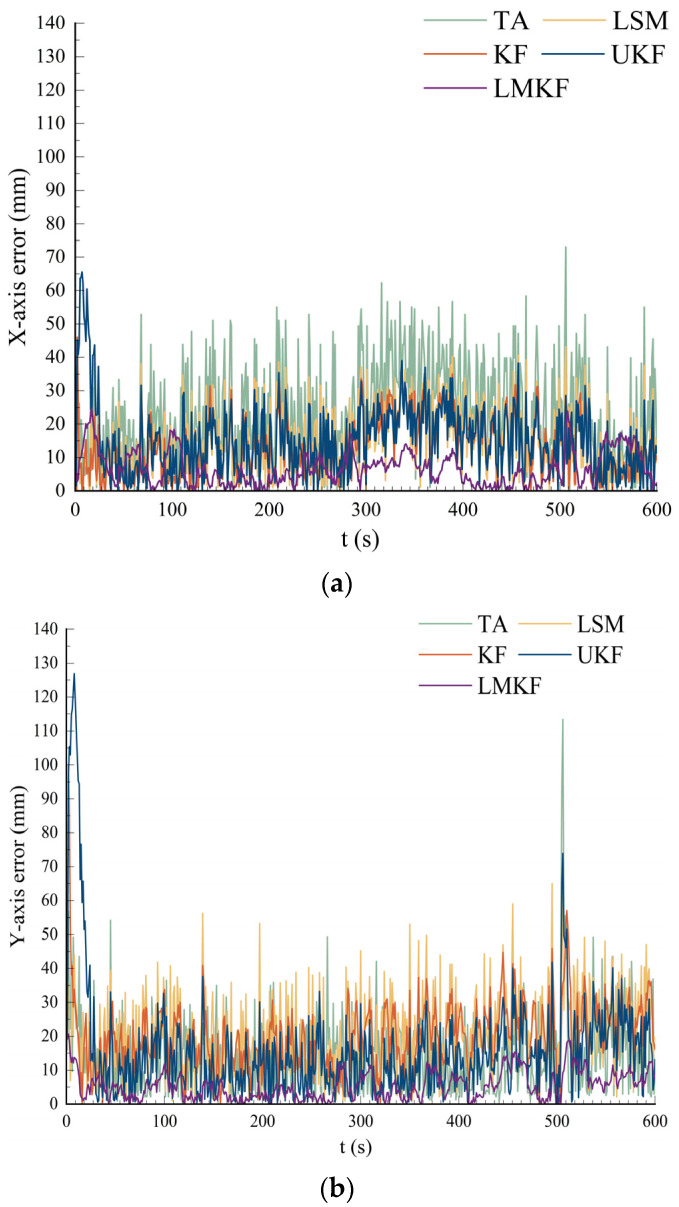
(**a**) X-axis error in LOS environment; (**b**) Y-axis error in LOS environment.

To verify the localization performance of the improved Kalman filtering algorithm proposed in this paper in a LOS environment, the experimental setup was set up as shown in Figure 7. The experimental location was North 505 of the School of Geomatics of Anhui University of Science and Technology. The coordinates of the base stations were set to be BS_A (0,0), BS_B (6,0), BS_C (6,5.4), and BS_D (0,5.4), and the coordinates of the labels were set to be Tag (3.6,2.4). The distance between the tag and the base station was determined using the ADS-TWR ranging algorithm. TA, LSM, KF, UFK, and LMKF were used to produce the positioning results, the X-axis errors, and the Y-axis errors, as shown in Figure 8 and Figure 9.

Figure 8 and Figure 9 show that the LMKF algorithm proposed in this paper exhibited significantly improved positioning accuracy compared to the other three positioning algorithms. The positioning coordinates obtained by the LMKF algorithm were more concentrated near the actual tag coordinates. In contrast, the positioning coordinates obtained by TA and LSM were more scattered. The positioning coordinates of the KF and UKF were similar to those of the LMKF algorithm, but several positioning coordinates were severely distorted and could not meet the positioning requirements. Positioning errors on the X axis and the Y axis were significantly reduced. The AVE and RMSE of the different algorithms are shown in Table 3.

According to Table 3, compared to the TA, LSM, KF, and UKF, the LMKF algorithm had a lower AVE in the X-axis direction by 70.20%, 54.12%, 48.99%, and 54.98%; in the Y-axis direction, it was reduced by 60.99%, 73.88%, 73.06%, and 54.98%. The RMSE was reduced by 66.81%, 63.83%, 60.78%, and 63.93%. The positioning results were significantly improved, demonstrating high positioning accuracy.

#### 4.2.3. Performance Analysis of Localization Algorithm in NLOS Environment

**Figure 10:**
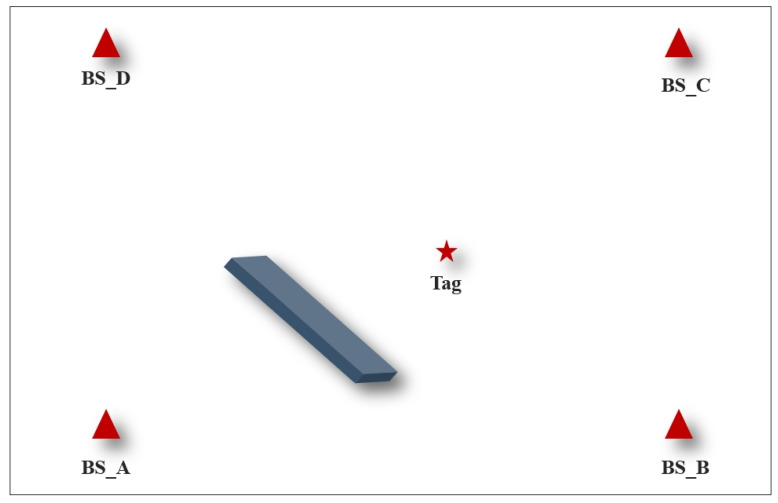
UWB base station and tag distribution map in NLOS environment.

**Figure 11:**
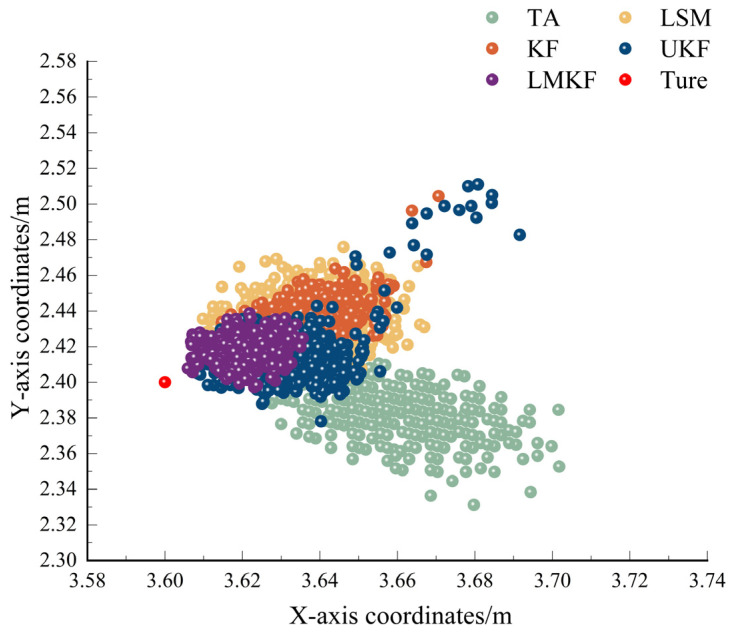
Scatter plot of localization in NLOS environment.

The signal propagation path is not a straight line because there are many obstacles in the actual localization process. It needs to be propagated through other paths such as reflection, refraction, etc., in which case the accuracy of the UWB localization algorithm is greatly affected. Therefore, to analyze the performance of the positioning algorithm proposed in this paper in an NLOS environment, based on the above experiments, the equipment arrangement was as shown in Figure 10. Obstacles were set up between BS_A and Tag to block the signal’s linear propagation between BS_A and Tag. The coordinates of the base station in the experiment were BS_A (0,0), BS_B (6,0), BS_C (6,5.4), and BS_D (0,5.4), and the coordinates of the tag were Tag (3.6, 2.4). The results of the five localization algorithms are shown in Figure 11.

**Figure 12:**
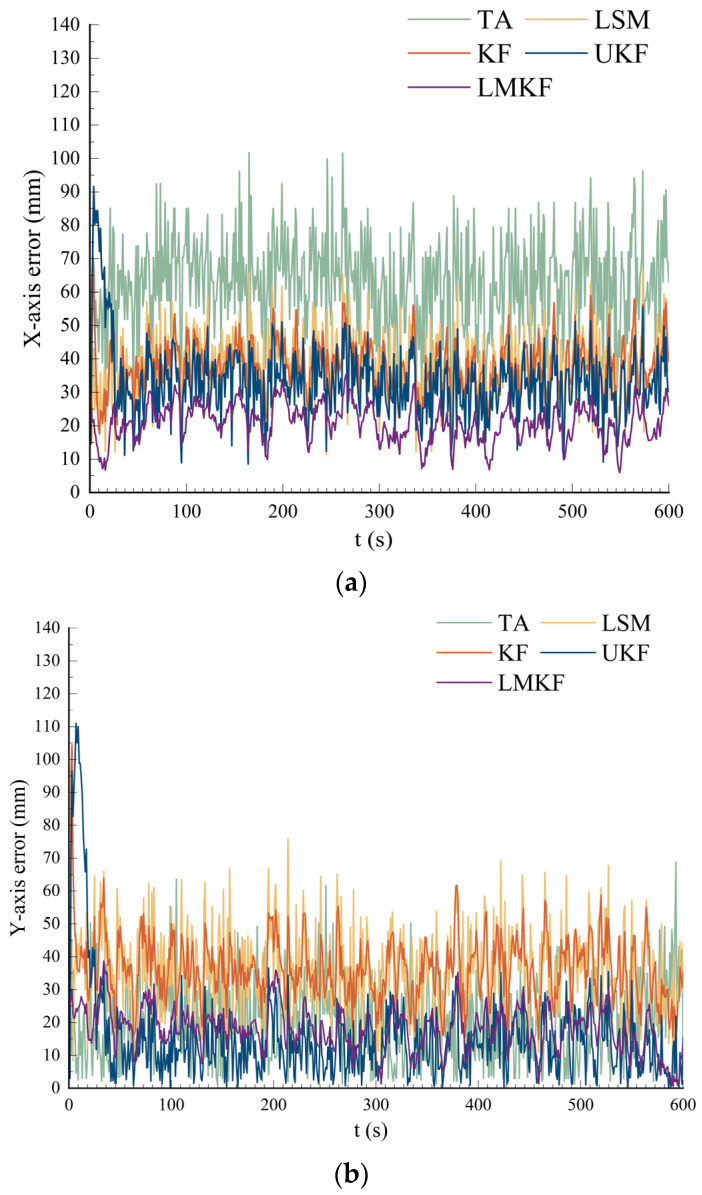
(**a**) X-axis error in NLOS environment; (**b**) Y-axis error in NLOS environment.

Due to the obstacles in the experiment, the signal propagation path between BS_A and Tag was not straight, resulting in a signal propagation time that was longer than the ideal propagation time. This caused the calculated distance between Tag and BS_A to be larger than the actual distance, which caused the localization results of the five algorithms to be biased to the upper right concerning the true position of the tag. Figure 12 shows that TA was most affected by NLOS, and the localization results of LSM, KF, and UKF were better than those TA, but a few points reflected the actual position of the tag. The localization results obtained by the LMKF were better than the other three methods, with more concentrated coordinates closer to the actual tag coordinates. The localization errors of the different algorithms are shown in Figure 12 and Table 4.

Figure 12 and Table 4 show that, compared with the LOS environment, the LMKF localization accuracy decreased significantly when in the NLOS environment, but the average localization error could still be controlled within about 20 mm in the NLOS environment. The LMKF can handle the non-line-of-sight error better than the TA, LSM, KF, and UKF. Compared with the TA, LSM, and KF, both the AVE and RMSE were significantly reduced, and the positioning accuracy was improved considerably. Compared with the UKF, although the AVE in the Y-axis direction was slightly increased, the AVE in the X-axis was significantly decreased, and the RMSE was reduced by 29.87%, indicating the proposed method can effectively overcome the slow convergence speed in the early stage of UKF positioning and has better stability.

## 5. Discussion

The LMKF algorithm proposed in this paper uses the ADS-TWR algorithm to obtain the distance from the UWB tag to each base station, and LSM and the KF are used to compute the tag’s location. However, due to the iterative nature of the observation equation of the KF, which is similar to the Gauss–Newton method of finding the extreme points of a function, its performance is often unstable. The state observation does not guarantee the reduction in the estimation error in the case where the covariance estimation is too low. The Levenberg–Marquardt algorithm is an excellent nonlinear optimization algorithm, which has fast global and local convergence. Therefore, the Levenberg–Marquardt algorithm was chosen to correct the covariance matrix of the KF, and the improved Kalman filtering algorithm was used to filter the initial position obtained by LSM to obtain the final position of the label. The experimental results showed that the tag position obtained by this algorithm was closer to the real position than those obtained by the other algorithms. Compared with the current common positioning algorithms, it has higher positioning accuracy and stability and faster convergence speed than the Kalman filter algorithm. In addition, the structure of the algorithm is relatively simple, has obvious advantages in terms of computational complexity and time complexity, does not require a large amount of a priori data, can be obtained by the device ranging value directly for positioning, and has a wider range of application scenarios in a variety of complex scenes.

The LMKF reduces the positioning error by improving the filtering algorithm, but some defects remain. The algorithm has good localization performance, but specific experiments in future work must still verify its performance in dynamic environments. At the same time, in the NLOS state, the signal propagation path is blocked by obstacles, which makes the actual signal propagation time longer than the signal propagation time in the ideal state, resulting in a significant increase in the ranging value compared to the actual value. The algorithm proposed in this paper does not have a method of identifying the LOS/NLOS state, which leads to a significant decrease in the localization effect in the NLOS state. Therefore, when the base station is deployed, it should be located in an area with no obstacles. As much as possible, to avoid obscured obstacles, future studies can add a LOS/NLOS state recognition algorithm, distinguish the LOS/NLOS state, and the NLOS state’s obtained ranging value can be used to correct to improve the algorithm’s positioning accuracy in complex environments.

## 6. Conclusions

An LMKF based UWB positioning method was proposed in this paper, focusing on the following aspects:

(1) Utilizing the TOF based ADS-TWR algorithm, distance information between UWB tags and base stations can be collected without strict clock synchronization. An LMKF algorithm was proposed, where the positioning results obtained from LSM serve as the initial position. The KF is then enhanced using the Levenberg–Marquardt algorithm, and the tag’s location is determined using the LMKF method.
(2) A series of simulation experiments were conducted. First, the UWB tag’s distances from each base station were calculated. To replicate the real data gathered, noise was applied to the distance information. Subsequently, the TA, LSM, KF, UKF, and the proposed LMKF algorithm were used to compute the tag coordinates. The experimental results demonstrated that the proposed LMKF algorithm achieved significantly lower AVE and RMSE under static conditions. Moreover, it effectively addressed the slow convergence issue of the KF and UKF in the initial stage. By varying the noise variance, it was proven that the proposed LMKF algorithm outperformed the TA, LSM, KF, and UKF under different noise conditions. The algorithm exhibited insensitivity to noise and maintained good performance even in high-noise environments, making it suitable for applications in severely noisy environments.
(3) A UWB localization system was constructed. The improved Kalman filter algorithm was experimentally verified in LOS and NLOS environments. The results showed that in the LOS environment, the average localization errors on the X axis and the Y axis were 6.8 mm and 5.4 mm, and the RMSE was 10.8 mm. In the NLOS environment, the AVE on the X axis and the Y axis were 20. 8 mm and 18.0 mm, and the RMSE was 28.9 mm. Compared with those of the TA, LSM, KF, and UKF, the positioning accuracy of LMKF was significantly higher, and the positioning coordinates was better concentrated near the actual tag coordinates, which proved that the UWB positioning algorithm proposed in this paper is highly accurate and stabile and has broad application prospects.

## Figures and Tables

**Table 1:** Error comparison of several algorithms.

Positioning Algorithm	AVE/mm		RMSE/mm
	X-Axis	Y-Axis	
TA	8.293	8.280	14.828
LSM	6.093	5.975	10.350
KF	5.360	5.780	13.901
UKF	13.316	16.435	36.423
LMKF	3.077	3.007	5.500

**Table 2:** MAX5007 module specifications.

Parameter	MAX5007
Dimension	33 × 13 × 2.9 mm
Frequency band	6.0–7.0 GHz
Coverage radius	1000 m
Ranging error	<10 cm
Positioning error	<15 cm

**Table 3:** Error comparison of different algorithms.

Positioning Algorithms	AVE/mm		RMSE/mm
	X Axis	Y Axis	
TA	22.809	13.873	32.581
LSM	14.813	20.723	29.895
KF	13.324	20.085	27.570
UKF	15.094	16.173	29.976
LMKF	6.795	5.411	10.813

**Table 4:** Error comparison of different algorithms in NLOS environment.

Positioning Algorithm	AVE/mm		RMSE/mm
	X-Axis	Y-Axis	
TA	62.665	20.860	68.595
LSM	37.481	35.980	54.572
KF	37.684	36.278	53.849
UKF	33.240	15.545	41.143
LMKF	20.779	18.020	28.852

## Data Availability

Dataset available upon request from the authors.
